# The hsa_circRNA_102049 mediates the sorafenib sensitivity of hepatocellular carcinoma cells by regulating *Reelin* gene expression

**DOI:** 10.1080/21655979.2021.2024332

**Published:** 2022-01-16

**Authors:** Shaolei Wang, Dehua Liu, Hong Wei, Yang Hua, Guodong Shi, Jinhan Qiao

**Affiliations:** Department of Medical Imaging, Cancer Hospital of China Medical University, Liaoning Cancer Hospital & Institute

**Keywords:** Hepatocellular carcinoma, sorafenib, hsa_circRNA_102049, Reelin, hsa-miR-214-3p, drug-resistance

## Abstract

A growing body of research has illuminated that non-coding RNAs (ncRNAs) plays an important role in the development of drug resistance in hepatocellular carcinoma (HCC) cells. The expression profiles of differential expressed genes (DEGs) and ncRNAs related to the sorafenib resistance in HCC cells were analyzed according to the Gene Expression Omnibus (GEO) dataSets and The Cancer Genome Atlas (TCGA) datasets. Bioinformatics technology was used to construct the interaction network of DEGs and ncRNAs. Cell transfection, dual-luciferase reporter assay, Western blot, cell counting kit-8 (CCK-8), flow cytometry and quantitative real-time polymerase chain reaction(qRT-PCR) were used to study the mechanism of sorafenib resistance in HepG2 cells and Huh-7 cells. The expression of reelin (RELN) and secretagogin (SCGN) were the only down-regulated in sorafenib-resistant HCC cells. The results showed that *RELN* gene demethylation reversed the cytotoxic of sorafenib on HepG2 cells and Huh-7 cells. Hsa_circRNA_102049 over-expression promoted the sensitivity of HepG2 cells and Huh-7 cells to sorafenib, hsa_circRNA_102049 up-regulated the expression of *RELN* gene by sponging hsa-miR-214-3p. The resistance to sorafenib in RELN knockout HepG2 cells and Huh-7 cells could be reverted by has-circRNA_102049. These findings support targeting of hsa_circRNA_102049 and RELN in sorafenib-treated HCC cells as a novel intervention, which is expected to overcome sorafenib resistance of HCC cells.

## GRAPHICAL ABSTRACT

**Figure:**
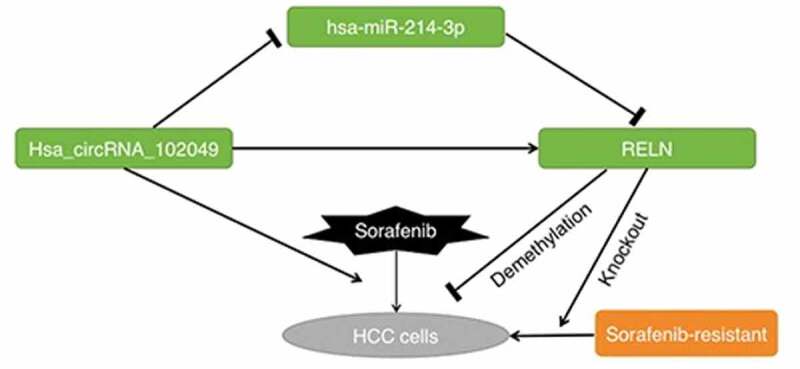

## 1. Introduction

Primary liver cancer is the fourth most common malignant tumor in China and the third leading cause of death among malignant tumors [1,2], of which hepatocellular carcinoma (HCC) accounts for 85%-90% [3]. Early-stage HCC can be treated with surgical resection, liver transplantation, and ablation [4], while curative treatment programs have limited effects on advanced HCC, and chemotherapy or local treatment is required [3].

Sorafenib is a tyrosine kinase inhibitor (TKI), which is currently the only effective molecular targeted drug approved by the US Food and Drug Administration for the treatment of advanced liver cancer [5]. Sorafenib can target Raf kinase and block the Ras/Raf/mitogen-activated protein kinases (MAPK) pathway and directly inhibit tumor cell proliferation, it can inhibit the activity of multiple receptors such as vascular endothelial-derived growth factor (VEGF) −2 and VEGFR receptor (VEGFR)-3 to inhibit tumor angiogenesis, and play a dual role of anti-tumor cell proliferation and anti-angiogenesis [6]. The results of a recent phase III clinical trial using another TKI, Lenvatinib for the treatment of advanced HCC were very encouraging. This study confirmed that Lenvatinib was not inferior to sorafenib in the treatment of advanced HCC [7]. However, studies have shown that the secondary drug resistance caused by long-term low-dose sorafenib exposure can enhance the migration and invasion of HCC cells [8,9]. The emergence of drug resistance in patients with advanced HCC after sorafenib treatment will reduce their survival and cause serious adverse reactions [10]. Therefore, studying the potential mechanism of sorafenib resistance is a necessary prerequisite for solving sorafenib resistance in advanced liver cancer patients.

Circular RNA (circRNA) is a type of ncRNA with a circular structure formed by covalent bonds [11,12]. In recent years, studies have shown that circRNAs have become a new favorite in the early diagnosis and prognosis evaluation of HCC [13,14]. For example, Li et al [15]. found that the circFBXO11/miR-605/forkhead box O3 (FOXO3)/ATP binding cassette transporter 1 (ABCB1) axis played an important role in the oxaliplatin resistance in HCC. Xu et al [16]. proposed that by targeting circRNA SORE (circRNA_104797) or YBX1 may be an effective solution to sorafenib resistance in HCC patients.

Therefore, this study aims to study the mechanism of hsa_circRNA_102049 on the sorafenib resistance of HCC cells, and provide new insights for solving the sorafenib resistance of HCC.

## 2. Materials and methods

### 2.1. DataSets

In this study, we obtained the research results from public databases. Among them, the differential gene expression data in HCC cancer tissues and adjacent tissues were obtained from the TCGA (https://portal.gdc.cancer.gov/). We selected 3 dataSets related to sorafenib resistance from the GEO (https://www.ncbi.nlm.nih.gov/gds/), namely GSE62813 dataSet, GSE94550 dataSet, and GSE73571 dataSet.

### 2.2. Cell culture

HepG2 cells, Huh-7 cells and HEK-293 T cells were obtained from the Cell Bank of Chinese Academy of Sciences (Beijing, China). Cells were cultured in a dulbecco’s modified Eagle’s medium (DMEM, Gibco) with a 1:1 ratio of penicillin and streptomycin (100 U/mL) and 10% fetal bovine serum (FBS, Gibco) [17]. Sorafenib (200 mg, tablets, Bayer Inc., Germany) was used to construct the sorafenib-resistant HepG2 cell line (namely SR-HepG2 cells) and Huh-7 cell line (namely SR-Huh-7 cells) by continuous induction.

### 2.3. Quantitative real-time polymerase chain reaction(qRT-PCR)

Total RNA was extracted from cells using TRIzol reagent (Invitrogen, Karlsruhe, Germany), and then reverse transcribed into cDNA by TAKARA PrimeScript Kit (Takara, Dalian, China). QRT-PCR was used to detect the relative expression levels of circRNAs and microRNA (miRNA), and glyceraldehyde-3-phosphate dehydrogenase (GAPDH) or β-actin was set as an internal control. The relative expression levels of circRNAs and miRNAs were detected by 2^−ΔΔct^ method [18]. The primer sequences were shown in Supplementary Table 1.

### 2.4. Cell transfection

Hsa_circRNA_102049 over-expression plasmid, hsa_circRNA_102049 small interfering RNA (si_circRNA_102049_1 and si_circRNA_102049_2), hsa-miR-526b-5p mimic, hsa-miR-214-3p mimic, hsa-miR-3619-5p mimic were purchased from GENEWIZ (Suzhou, China). All sequence information was shown in Supplementary Table 1. Oligonucleotides and constructs were transfected into the HepG2 cells and Huh-7 cells using Lipofectamine 2000 (Invitrogen, Carlsbad, CA) according to the supplier’s instructions, and the empty vector was set as the control [19].

### 2.5. Dual-luciferase reporter assay

The dual-luciferase reporter assay was used to detect the interaction between miRNAs and circRNAs or target gene. The hsa_circRNA_102049 wild-type (WT) or mutant (MUT), *RELN* 3ʹUTR wild-type (wt) or mutant (mut) were cloned into pGL3-Promoter Vector (Promega), and pGL3-hsa_circRNA_102049 WT or pGL3-hsa_circRNA_102049 MUT with hsa-miR-526b-5p mimic, hsa-miR-214-3p mimic, hsa-miR-3619-5p mimic or empty vector (Control) were transfected into HET-293 T cells using Lipofectamine 2000 (Invitrogen, Carlsbad, CA). The pGL3-RELN wt or pGL3-RELN mut and hsa-miR-214-3p mimic or Control were transfected into HET-293 T cells. The dual-luciferase assay system (Promega) was used to detect and analyze the luciferase activity [20].

### 2.6. Western blot

Radioimmunoprecipitation assay (RIPA) lysis buffer was used to extract total proteins from cultured HepG2 cells and Huh-7 cells, and bicinchoninic acid (BCA) assay was used for proteins quantification. Then sodium dodecyl sulfate polyacrylamide gel electrophoresis (SDS-PAGE) assay was used to separate the proteins, then proteins were transferred to polyvinylidene fluoride (PVDF) membrane and seal with skim milk for 1–2 h at room temperature. Then the primary antibody (Anti-Reelin, 1:1000, #ab139691, Abcam, UK; Anti-N Cadherin, 1:5000, #ab76011, Abcam, UK; Anti-E Cadherin, 1:500, #ab40772, Abcam, UK) was added and incubated overnight (12–16 h) at 4°C, and β-actin served as an internal control. The relative gray value of the target protein and β-actin was calculated [21].

### 2.7. Cell counting Kit-8

The cultured HepG2 cells and Huh-7 cells were seeded in a 96-well plate at a density of 1 × 10^3^ cells/well, and the cell proliferation viability was detected using Cell Counting Kit-8 (CCK-8, Dojindo, Japan) after 72 hours of culture. Cell proliferation rate = (treatment group – blank cell-free group)/(control group/blank cell-free group) * 100% [22].

### 2.8. Apoptosis detection

Flow cytometry was used to detected the apoptosis rate of HepG2 cells and Huh-7 cells. After 48 hours of transfection, Annexin V-fluorescein isothiocyanate (FITC) and propidium iodide (PI) double staining kit (Becton Dickinson, USA) were used to detect the apoptosis rate [17].

### 2.9. Gene differential expression analysis

The RStudio software (Version 1.3.1073) was used to study the differential expression of mRNAs. The analysis method of related gene function can be found in this research [23].

### 2.10. Statistical analysis

In this study, all groups were made in triplicate, and the data were expressed in mean ± Standard deviation. Graphpad prism version 8.0 for Mac (GraphPad Software, USA) was used for statistical analysis. The statistical difference between the groups was performed by *t* test or one-way analysis of variance (ANOVA). All tests were two-tailed, and *p* < 0.05 indicated a statistical difference.

## 3. Results

This study aims to analyze the mechanism of action of non-coding RNA in the process of sorafenib resistance in HCC cells. Based on the data obtained in GEO dataSets and TCGA, the differential gene and ncRNAs expression profiles related to the occurrence of HCC and sorafenib resistance were analyzed. Then bioinformatics technology was used to construct the differentially expressed genes and ncRNAs interaction network. Sorafenib-resistant HepG2 cells and Huh-7 cells were constructed respectively. Cell transfection, dual-luciferase reporter assays, Western blot, cell counting kit-8 and flow cytometry were used to analyze the mechanism of circular RNA hsa_circRNA_102049 in the process of sorafenib resistance in HCC cells.

### 3.1. Analysis of differential expression gene in HCC

**Figure 1.**
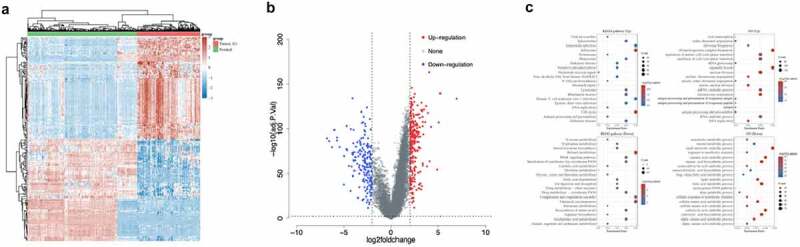
Analysis of differentially expressed genes in HCC tissues and normal tissues. a. Heat map of differentially expressed genes. The different colors represent expression trends in different tissues. Due to the large number of differential genes, the 50 up-regulated genes and 50 down-regulated genes with the largest differential changes were shown here. b. Volcano plots were constructed using fold-change values and adjusted P. The red points in the figure indicated genes that were significantly up-regulated, and blue points indicated genes that were significantly down-regulated. c. The enriched KEGG signaling pathways were selected to demonstrate the primary biological actions of major potential mRNAs. The abscissa indicated gene ratio and the enriched pathways were presented in the ordinate. Gene ontology (GO) analysis of potential targets of mRNAs. The biological process (BP), cellular component (CC), and molecular function (MF) of potential targets were clustered based on ClusterProfiler package in R software (version: 3.18.0). In the enrichment result, p < 0.05 or FDR <0.05 was considered to be enriched to a meaningful pathway.

A total of 158 Asian HCC patients’ information was selected from the TCGA database. The clinical data were shown in Table 1. The results showed that during the occurrence of HCC, a total of 184 genes were up-regulated (log2 fold change>2, adj.P.Val<0.01), and 151 genes were down-regulated (log2 fold change<-2, adj.P.Val<0.01) (Supplementary Table 2, Figure 1(a,b)). The up-regulated genes were mainly related to ribonucleoprotein complex biogenesis, etc., while genes related to carboxylic acid biosynthetic process, etc. were down-regulated (Figure 1(c)).

**Table 1.** Clinical data of patients with HCC

Parameter	Characteristic	HCC(N = 158)
Status	Alive	114
	Dead	44
Age	Mean (SD)	55.2 (11.6)
	Median (min, max)	56 (18, 76)
Gender	female	34
	male	124
Race	Asian	158
pT_stage	T1	80
	T2	35
	T3	24
	T3a	13
	T4	6
pN_stage	N0	146
	N1	1
	NX	11
pM_stage	M0	150
	M1	1
	MX	7
pTNM_stage	I	79
	II	35
	IIIA	35
	IIIB	3
	IIIC	5
	IV	1
Grade	G1	14
	G2	64
	G3	69
	G4	11

HCC, hepatocellular carcinoma. SD, Standard deviation.

### 3.2. Analysis of differentially expressed genes in sorafenib-resistant HCC cells

**Figure 2.**
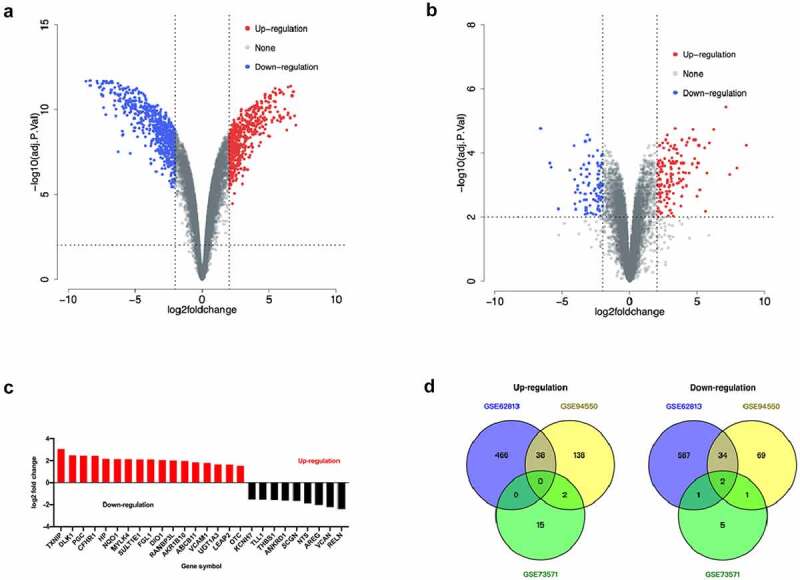
Analysis of differentially expressed genes in sorafenib-resistant HCC cells. a. Volcano map of the gene expression related to sorafenib resistance in the GSE62813 dataSet. b. Volcano map of gene expression related to sorafenib resistance in the GSE94550 dataSet. c. Volcano map of gene expression related to sorafenib resistance in the GSE73571 dataSet. d. Venn diagram of sorafenib resistance-related gene expression in GSE62813, GSE94550 and GSE73571 dataSets.

There were 526 genes were up-regulated and 657 genes were down-regulated according to the GSE62813 dataSet (Supplementary Table 3 and Figure 2(a)). Among them, 179 genes related to sorafenib resistance in the GSE94550 dataSet were up-regulated and 106 genes were down-regulated (Supplementary Table 4 and Figure 2(b)). A total of 17 genes related to sorafenib resistance in the GSE73571 dataSet were up-regulated and 9 genes were down-regulated (Supplementary Table 5 and Figure 2(c)). Only *RELN* and *SCGN* gene were significantly down-regulated in sorafenib-resistant HCC cells according to these three dataSets (Figure 2(d)).

**Figure 3.**
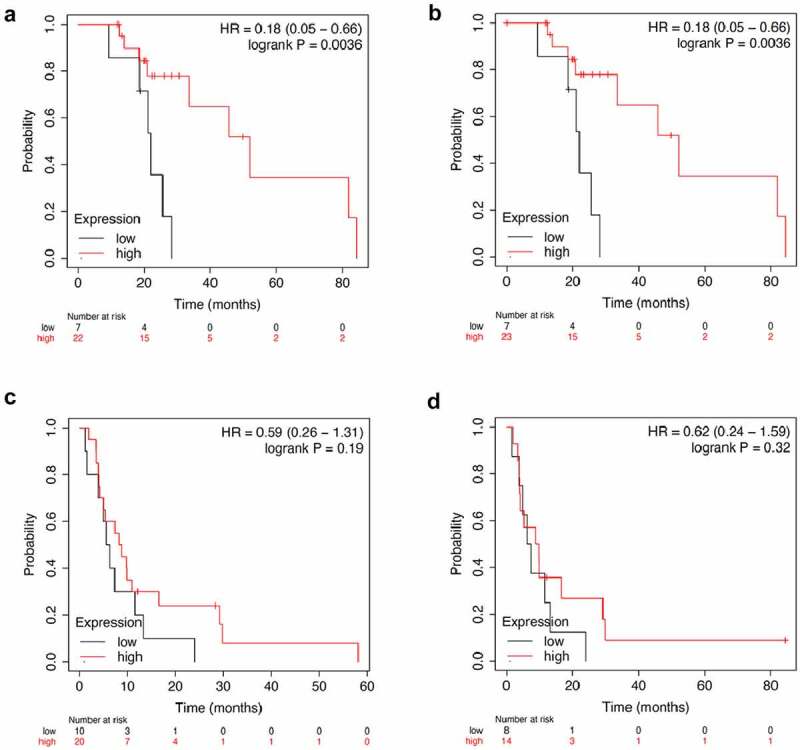
Analysis of *RELN* gene expression and survival of HCC patients. a. The correlation between *RELN* gene expression and overall survival (OS) of HCC patients. b. The correlation between *RELN* gene expression and disease-free survival (DFS) of HCC patients. C. The correlation between *RELN* gene expression and progression free survival (PFS) of HCC patients. d. The correlation between *RELN* gene expression and recurrence free survival (RFS) of HCC patients.

The expression of *SCGN* gene in HCC tumor tissues was significantly up-regulated (log2 fold change = 1.88, p < 0.01), while the expression level of *RELN* gene in HCC tumor tissues did not change significantly (log2 fold change = 0.84, p = 0.55). Among the HCC patients treated with sorafenib, the Overall Survival (OS) and disease free survival (DFS) of HCC patients with low *RELN* expression were significantly lower than those of patients with high *RELN* expression (p = 0.0036, Figure 3(a); p = 0.0036, Figure 3(b)), there were no significant differences in progression free survival (PFS) and recurrence free survival (RFS) between HCC patients with low and high *RELN* expression (p = 0.19, Figure 3(c); p = 0.32, Figure 3(d)). We believed that the expression of *RELN* gene was related to the prognosis of HCC patients after sorafenib treatment. Therefore, we chose the RELN gene for further research.

### 3.3. *Demethylation of* RELN *gene reversed the cytotoxic effect of sorafenib on HCC cells*

**Figure 4.**
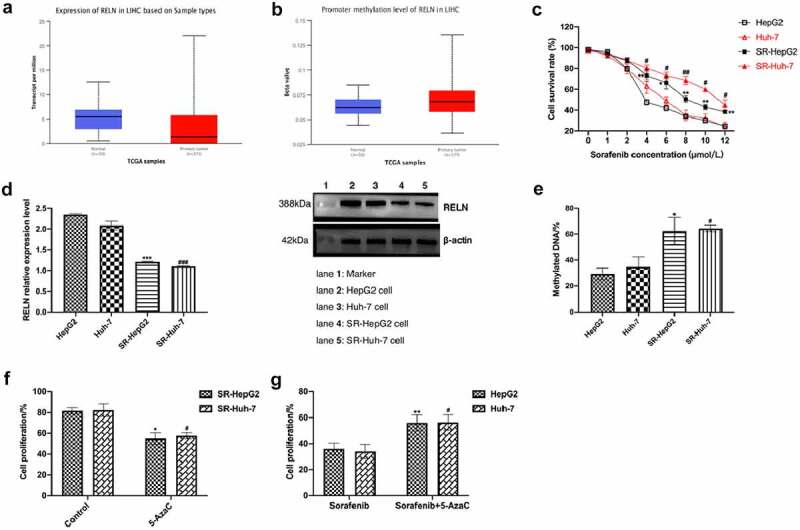
*RELN* gene demethylation reversed the cytotoxic effect of sorafenib on HCC cells. a. RELN was low expressed in HCC tumor tissues, p < 0.001. b. The methylation level of *RELN* gene promoter region in HCC tumor tissue was higher than that in normal tissue, p < 0.001. c. The cell proliferation ability of HepG2, Huh-7, SR-HepG2, SR-Huh-7 cells treated with different concentrations of sorafenib were detected by Cell Counting Kit-8 (CCK-8). *p < 0.05, **p < 0.01, compared to HepG2. #p < 0.05, ##p < 0.01, compared to Huh-7. D. The expression level of RELN protein in HepG2, Huh-7, SR-HepG2, SR-Huh-7 cells were detected by Western blot. ***p < 0.001, compared to HepG2. ###p < 0.001, compared to Huh-7. E. Comparison of the *RELN* gene promoter methylation level between HepG2, Huh-7, SR-HepG2, SR-Huh-7 cells. *p < 0.05, compared to HepG2. #p < 0.05, compared to Huh-7. F. The cell proliferation ability of SR-HepG2 and SR-Huh-7 cells after 5-AzaC treatment were detected by CCK-8 assay. *p < 0.05, compared to HepG2. #p < 0.05, compared to Huh-7. G. The cell proliferation ability of HepG2 cells and Huh-7 cells treated with sorafenib, sorafenib+5-AzaC were detected by CCK-8 assay. *p < 0.05, compared to HepG2. #p < 0.05, compared to Huh-7.

To analyze the role of the *RELN* gene in sorafenib-resistant HCC cells, we analyzed the drug gene interaction database (https://dgidb.genome.wustl.edu/search_interactions) and found that there was no interaction between *RELN* gene and sorafenib (Supplementary Table 6), so we believed that *RELN* was not a direct target of sorafenib. We used the UALCAN tool to study the expression level and methylation level of genes in TCGA samples and found that *RELN* was highly methylated in HCC tumor tissues, and the *RELN* gene expression level was significantly lower than that of normal tissues (Figure 4(a,b)). We constructed sorafenib-resistant HepG2 cell lines (namely SR-HepG2 cells) and Huh-7 cell lines (namely SR-Huh-7 cells) by sorafenib stimulation (Figure 4(c)).

Next, we analyzed the expression level and methylation level of RELN in SR-HepG2 cells and SR-Huh-7 cells. The results showed that the expression level of RELN in SR-HepG2 cells and SR-Huh-7 cells were lower than those in HepG2 cells and Huh-7 cells (Figure 4(d)), and the methylation level were significantly higher than those in HepG2 cells and Huh-7 cells (Figure 4(e)). After 5-AzaC treated, the proliferation of SR-HepG2 cells and SR-Huh-7 cells were significantly inhibited (Figure 4(f)), and the inhibition of cell proliferation in HepG2 and Huh-7 cells treated with sorafenib was reversed by 5-AzaC (Figure 4(g)), suggesting that the *RELN* gene demethylation reversed the cytotoxic effects of sorafenib on HepG2 cells and Huh-7 cells.

### 3.4. Analysis of ncRNAs in sorafenib-resistant HCC cells

**Figure 5.**
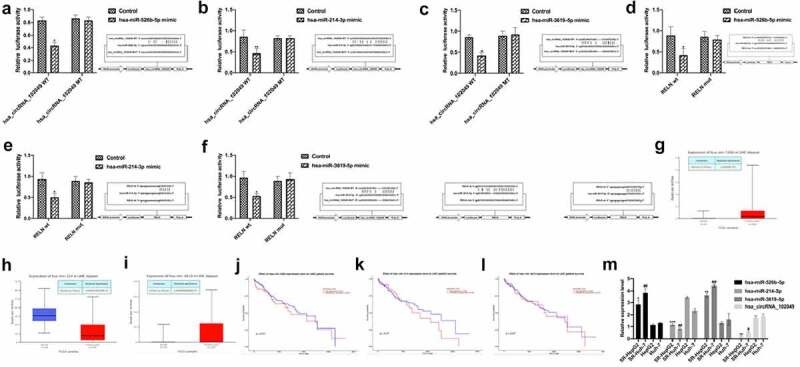
The expression of ncRNAs in sorafenib-resistant HCC cells. A-C. The predicted binding sites of hsa_circRNA_102049 and hsa-miR-526b-5p (a), hsa-miR-214-3p (b), hsa-miR-3619-5p (c) and the designed binding site mutation sequences. Comparison of the relative luciferase activity of cells co-transfected with hsa_circRNA_102049 WT or MUT and hsa-miR-526b-5p mimic (a), hsa-miR-214-3p mimic (b), hsa-miR-3619-5p mimic (c) or Control. Data was expressed as mean ± standard deviation (n = 3, each group), *p < 0.05, **p < 0.01, compared to the control. The predicted *RELN* 3ʹ UTR and hsa-miR-526b-5p (d), hsa-miR-214-3p (e), hsa-miR-3619-5p (f) binding site and the designed binding site mutation sequence. Comparison of the relative luciferase activity of cells co-transfected with RELN wt or mut and hsa-miR-526b-5p mimic (D), hsa-miR-214-3p mimic (E), hsa-miR-3619-5p mimic (f) or Control. Data was expressed as mean ± standard deviation (n = 3, each group), *p < 0.05, compared to the control. Comparison of the expression of hsa-miR-526b-5p (g), hsa-miR-214-3p (h), hsa-miR-3619-5p (i) between HCC tissues and normal control samples in the TCGA sample database, data came from UALCAN (http:// ualcan.path.uab.edu/index.html). The expression level of hsa-miR-526b-5p (j), hsa-miR-214-3p (k), hsa-miR-3619-5p (l) and the survival of HCC patients, data came from UALCAN (http://ualcan.path.uab.edu/index.html). m. The expression level of hsa_circRNA_102049, hsa-miR-526b-5p, hsa-miR-3619-5p, hsa-miR-214- in SR-HepG2, SR-Huh-7, HepG2, and Huh-7 cells were detected by qRT-PCR.

We obtained the GSE101850 dataSet from the GEO dataSets and analyzed the differentially expressed circRNAs in sorafenib-resistant cells (Supplementary Table 7). Among them, the expression of hsa_circRNA_102049 (hsa_circ_0043278) was highest down-regulated. The prediction results by the Encyclopedia of RNA Interactomes (ENCORI) showed that hsa-miR-526b-5p, hsa-miR-214-3p, hsa-miR-3619-5p all had sequences that bound to hsa_circRNA_102049 and *RELN* 3ʹUTR (Figure 5(a-f), Supplementary Table 8). The results of the dual luciferase report assay showed that compared with the control group, the ectopic expression of hsa-miR-526b-5p mimic, hsa-miR-214-3p mimic, hsa-miR-3619-5p mimic and hsa_circRNA_102049 WT or RELN wt reduced the luciferase activities (Figure 5(a-f)). Interestingly, when analyzing the microRNAs expression data in TCGA samples, we found that hsa-miR-526b-5p and hsa-miR-3619-5p were highly expressed in HCC tissues, while hsa-miR-214-3p was low expressed in HCC tissues (Figure 5(g-i)), these differences in expression were not significantly related to patient survival (Figure 5(j-l)). The results of qRT-PCR analysis showed that the expression levels of hsa-miR-526b-5p and hsa-miR-3619-5p in SR-HepG2 and SR-Huh-7 were higher than those in HepG2 and Huh-7 cells, while the expression levels of hsa-miR-214-3p and hsa_circRNA_102049 were lower than HepG2 and Huh-7(Figure 5(m)). These results suggested that hsa_circRNA_102049, hsa-miR-526b-5p, hsa-miR-214-3p, hsa-miR-3619-5p were related to sorafenib resistance in HCC cells.

### 3.5. Hsa_circRNA_102049 over-expression promoted the sensitivity of HCC cells to sorafenib

**Figure 6.**
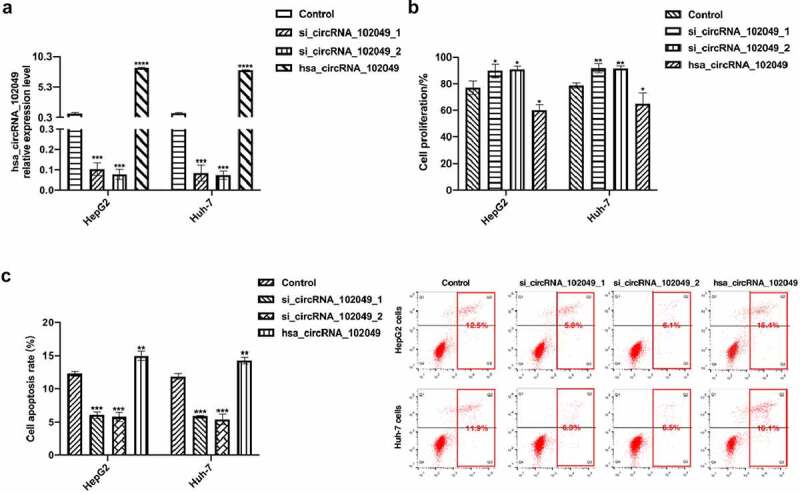
Hsa_circRNA_102049 over-expression promoted the sensitivity of HCC cells to sorafenib. a. The relative expression level of hsa_circRNA_102049 in HepG2 cells and Huh-7 cells were detected by qRT-PCR after control, si_circRNA_102049_1, si_circRNA_102049_2, hsa_circRNA_102049 were transfected into HepG2 and Huh-7 cells. b. The cell proliferation ability of sorafenib-treated HepG2 cells and Huh-7 cells after transfected with Control, si_circRNA_102049_1, si_circRNA_102049_2, hsa_circRNA_102049 were detected by Cell Counting Kit-8 (CCK-8). c. The apoptosis rate of HepG2 cells and Huh-7 cells transfected with Control, si_circRNA_102049_1, si_circRNA_102049_2, hsa_circRNA_102049 were detected by flow cytometry assay. *p < 0.05, **p < 0.01, ***p < 0.001, compared to the control.

In order to down-regulate the expression level of hsa_circRNA_102049, two kinds of hsa_circRNA_102049 small interfering RNAs were transfected into HepG2 cells and Huh-7 cells (Figure 6(a)). The hsa_circRNA_102049 over-expressed plasmid was also successfully transfected into HepG2 and Huh-7 cells (Figure 6(a)). After stimulation with sorafenib, the proliferation activity of hsa_circRNA_102049 silenced HepG2 cells and Huh-7 cells were significantly higher than those of hsa_circRNA_102049 over-expressed HepG2 cells and Huh-7 cells, and the apoptosis rate were significantly lower than those of hsa_circRNA_102049 over-expressed HepG2 cells and Huh-7 cells (Figure 6(b,c)). These results suggested that hsa_circRNA_102049 over-expression promoted the sensitivity of HCC cells to sorafenib.

### 3.6. *Hsa_circRNA_102049 up-regulated the expression of* RELN *gene by sponging hsa-miR-214-3p*

**Figure 7.**
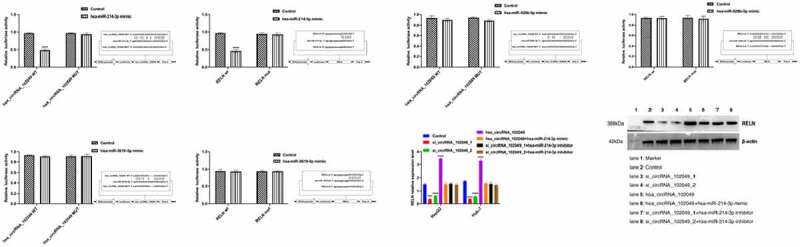
Hsa_circRNA_102049 up-regulated the expression of RELN by sponging hsa-miR-214-3p. a. Comparison of luciferase activity after hsa_circRNA_102049 WT+hsa-miR-214-3p mimic, hsa_circRNA_102049 MUT+hsa-miR-214-3p mimic, hsa_circRNA_102049 WT+Control, hsa_circRNA_102049 MUT+Control transfected into HEK-293 T cells. b. Comparison of luciferase activity after RELN wt+hsa-miR-214-3p mimic, RELN mut+hsa-miR-214-3p mimic, RELN wt+Control, RELN mut+Control transfected into HEK-293 T cells. C. Comparison of luciferase activity after hsa_circRNA_102049 WT+hsa-miR-526b-5p mimic, hsa_circRNA_102049 MUT+hsa-miR-526b-5p mimic, hsa_circRNA_102049 WT+Control, hsa_circRNA_102049 MUT+Control transfected into HEK-293 T cells. d. Comparison of luciferase activity after RELN wt+hsa-miR-526b-5p mimic, RELN mut+hsa-miR-526b-5p mimic, RELN wt+Control, RELN mut+Control transfected into HEK-293 T cells. e. Comparison of luciferase activity after hsa_circRNA_102049 WT+hsa-miR-3619-5p mimic, hsa_circRNA_102049 MUT+hsa-miR-3619-5p mimic, hsa_circRNA_102049 WT+Control, hsa_circRNA_102049 MUT+Control transfected into HEK-293 T cells. f. Comparison of luciferase activity after RELN wt+hsa-miR-3619-5p mimic, RELN mut+hsa-miR-3619-5p mimic, RELN wt+Control, RELN mut+Control transfected into HEK-293 T cells. g. The RELN expression level of HepG2 cells and Huh-7 cells transfected with Control, si_circRNA_102049_1, si_circRNA_102049_2, hsa_circRNA_102049, hsa_circRNA_102049+ hsa-miR-214-3p mimic, si_circRNA_102049_1+ hsa-miR-214-3p inhibitor, si_circRNA_102049_2+ hsa-miR-214-3p inhibitor. *p < 0.05, ****p < 0.0001, compared to the Control.

Based on the previous prediction research results of the Encyclopedia of RNA Interactomes (Supplementary Table 8), we conducted a dual luciferase assay. The results showed that the luciferase activity of hsa_circRNA_102049 wt+hsa-miR-214-3p mimic and RELN wt+hsa-miR-214-3p mimic were significantly reduced after transfection, however there were no significant changes in luciferase activity after hsa_circRNA_102049 mut+hsa-miR-526b-5p mimic, hsa_circRNA_102049 mut+hsa-miR-3619-5p mimic, RELN mut+hsa-miR-526b-5p mimic, RELN mut+hsa-miR-3619-5p mimic transfection (Figure 7(a-c)).

Then si_circRNA_102049_1, si_circRNA_102049_2, and hsa_circRNA_102049 were transfected into HepG2 and Huh-7 cells respectively (Figure 7(d)). The results showed that the expression level of RELN decreased significantly after hsa_circRNA_102049 was silenced, while increased significantly in hsa_circRNA_102049 up-regulated HepG2 and Huh-7 cells (Figure 7(e)). However, the expression of RELN in HepG2 and Huh-7 cells co-transfected with hsa_circRNA_102049 and hsa-miR-214-3p mimic were not significantly different compared to the control, the same results appeared in HepG2 cells and Huh-7 cells co-transfected with si_circRNA_102049_1 or si_circRNA_102049_2 and hsa-miR-214-3p inhibitor (Figure 7(f)). These results suggested that hsa_circRNA_102049 up-regulated the expression of RELN by sponging hsa-miR-214-3p.

### 3.7. *Hsa_circRNA_102049 reversed the sorafenib resistance of* RELN *knockout HCC cells*

**Figure 8.**
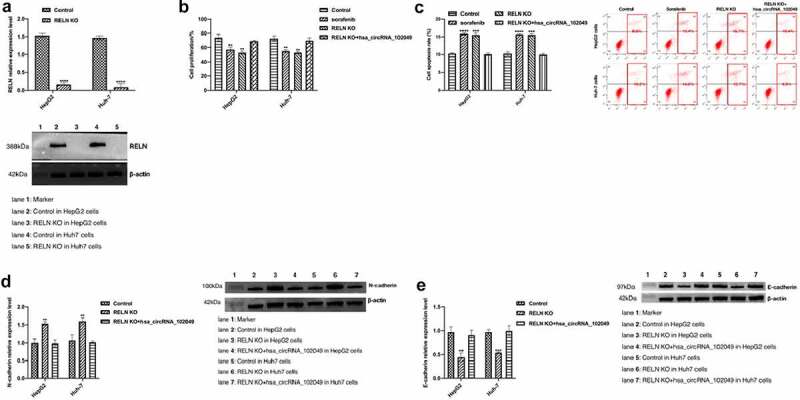
Hsa_circRNA_102049 reversed the sorafenib resistance of RELN knockout HCC cells. a. The expression of RELN in *RELN* gene knocking out HepG2 cells and Huh-7 cells were detected by Western blot b. The proliferation ability of HepG2 cells and Huh-7 cells transfected with Control, RELN knockout (KO), RELN KO+hsa_circRNA_102049 were detected by CCK-8 assay. c. The apoptosis rate of HepG2 cells and Huh-7 cells transfected with Control, RELN knockout (KO), RELN KO+hsa_circRNA_102049 were detected by flow cytometry. d. The expression level of RELN, N-cadherin and E-cadherin proteins in HepG2 cells and Huh-7 cells transfected with Control, RELN KO, RELN KO+hsa_circRNA_102049 were detected by Western blot. *p < 0.05, **p < 0.01, ***p < 0.001, compared to the Control.

We successfully knocked out the *RELN* gene in HepG2 and Huh-7 cells (Figure 8(a)). Compared with the control, the proliferation activity of the HepG2 cells and Huh-7 cells did not change significantly after sorafenib stimulation, but after transfection with hsa_circRNA_102049, the cell proliferation activity was significantly decreased, and the apoptosis rate was significantly increased (p < 0.05, Figure 8(b)). After knocking out the *RELN* gene, the expression of N-cadherin was significantly up-regulated and E-cadherin was significantly down-regulated, while after transfection with hsa_circRNA_102049, the expression of N-cadherin was significantly down-regulated, and E-cadherin was significantly up-regulated (Figure 8(c)). These results showed that hsa_circRNA_102049 reversed the sorafenib resistance of *RELN* knockout HCC cells.

## 4. Discussion

Research evidences have shown that circRNAs played an important role in the diagnosis and treatment of HCC. CircRNA microarray, qRT-PCR and other technical methods have been used to study the differential expression levels of circulating circRNAs or exosomal circRNAs in cancer tissues and peripheral blood of HCC patients. Screening HCC diagnostic markers and developing effective targets for HCC treatment have become research hotspots [14,24,25]. Researchers found that circRNAs played an important role in HCC cell proliferation, migration, invasion, apoptosis and drug resistance [26,27]. In this study, we found that the newly discovered hsa_circRNA_102049 played an key role in the sorafenib resistance of HCC cells.

In this study, we found that hsa_circRNA_102049 was significantly low expressed in sorafenib-resistant HepG2 cells and Huh-7 cells. After down-regulated the expression of hsa_circRNA_102049 by small interfering RNA, we found that HepG2 cells and Huh-7 cells were not sorafenib sensitive. The proliferation capacity was significantly inhibited and the apoptotic rate were increased after sorafenib stimulation in the hsa_circRNA_102049 overexpressed HepG2 cells and Huh-7 cells. These results suggested that overexpression of hsa_circRNA_102049 promoted the sorafenib sensitivity of HCC cells.

Although our findings proved that hsa_circRNA_102049 was associated with sorafenib resistance in HCC cells, the specific mechanism was still unclear. We found that the expression levels of RELN and SCGN were significantly down-regulated in in sorafenib-resistant HCC cells in GSE62813, GSE94550 and GSE73571 dataSet. We found that *SCGN* gene expression was up-regulated in HCC tumor tissues, but down-regulated in sorafenib-resistant HCC cells. This seemingly contradictory result reduced our interest in studying SCGN. The expression level of *RELN* gene in HCC tumor tissues did not change significantly, but it was down-regulated in sorafenib-resistant HCC cells. In addition, among HCC patients treated with sorafenib, the OS and DSS of patients with low RELN expression were significantly lower than those with high RELN expression. Therefore, we believed that the down-regulation of *RELN* gene expression may be related to the sorafenib resistance of HCC cells.

The *RELN* gene is located on the 7q22.1 chromosome and encodes a large number of secreted extracellular matrix proteins. These proteins are closely related to cell-to-cell interactions that involve cell localization and neuron migration during brain development [28]. Studies have reported that the *RELN* gene was a key gene related to the recurrence of HCC [29]. Some researchers have found that the *RELN* gene plays an important role in inhibiting the migration of HCC cells [30]. The mechanism may be that RELN induces epithelial-mesenchymal transition (EMT) to promote the HCC cells cycle process, migration and invasion [31]. In this study, we found that the *RELN* gene was highly methylated in HCC tumor tissues and sorafenib-resistant HCC cells, and the demethylation of the *RELN* gene reversed the effect of sorafenib on HCC cells. These results proved that *RELN* gene expression level affected the sorafenib sensitivity of HCC cells.

Based on the results of bioinformatics analysis and in vitro experiments, we found that the *RELN* gene was the target gene of hsa_circRNA_102049, and hsa_circRNA_102049 up-regulated the expression of *RELN* gene by sponging hsa-miR-214-3p. Especially when we knocked out the *RELN* gene in HCC cells, the sorafenib sensitivity of HCC cells was significantly reduced, and the sorafenib resistance of HCC cells was reversed by hsa_circRNA_102049. These results indicated that RELN and hsa_circRNA_102049 were potentially reliable targets for solving the sorafenib resistance of HCC cells. Further studies have found that hsa_circRNA_102049 targeted and regulated the expression of the *RELN* gene and solved the sorafenib resistance of HCC cells through the EMT pathway.

In recent years, research evidences have shown that circRNAs play an important role in the multi-drug resistance of cancer chemotherapeutics. For example, Li et al [15]. found that circFBXO11 regulated the oxaliplatin resistance of HCC cells by targeting the miR-605/FOXO3/ABCB1 axis. Xu et al [16]. found that circRNA-SORE mediated sorafenib resistance of HCC cells through targeted regulation of YBX1. Guan et al [32]. proved that circRNA_102272 regulated the cisplatin resistance of HCC cells by targeting RUNX2. This study also revealed the potential value of a new type of circRNA hsa_circRNA_102049 in solving the sorafenib resistance of HCC cells. Overexpression of hsa_circRNA_102049 promoted the sorafenib sensitivity of HCC cells. Mechanism studies have shown that hsa_circRNA_102049 can up-regulate the expression of *RELN* gene, mediate the apoptosis of sorafenib-resistant HCC cells through the EMT pathway, and solve the sorafenib resistance of HCC cells. However, it should be noted that our research needs to be further verified with in vivo studies.

## 5. Conclusion

In conclusion, our study has discovered a new type of circRNA hsa_circRNA_102049, which plays a key role in the sorafenib resistance of HCC cells. Hsa_circRNA_102049 up-regulates the expression of *RELN* gene to effectively solve the sorafenib resistance of HCC cells. It provides the possibility to effectively solve the sorafenib resistance in the advanced HCC patients.

## Supplementary Material

Supplemental Material

## Highlights

1. RELN and SCGN were down-regulated in sorafenib-resistant HCC cells.
2. *RELN* gene demethylation reversed the cytotoxic of sorafenib on HCC cells.
3. Hsa_circRNA_102049 over-expression promoted the sensitivity of HCC cells to sorafenib.
4. Hsa_circRNA_102049 up-regulated the expression of *RELN* gene by sponging hsa-miR-214-3p.
5. Hsa_circRNA_102049 reversed the resistance of *RELN* gene knockout HCC cells to sorafenib.
