# Supplementary material for: The hsa_circRNA_102049 mediates the sorafenib sensitivity of hepatocellular carcinoma cells by regulating Reelin gene expression
Source: Bioengineered. 2022 Jan 16;13(2):2272–84. doi: 10.1080/21655979.2021.2024332 (PMC8973865; doi:10.1080/21655979.2021.2024332)
Supplement: Supplemental Material [file KBIE_A_2024332_SM6432.zip › supplementary/supplementary table 1.doc]

Supplement Table 1. circRNA and miRNA sequence and qRT-PCR primer sequence.

| RNA ID | 5’-3’ |
| --- | --- |
| hsa_circRNA_102049 | ACTTCAGATTTTCCTGTCCTTGATCCCAGCTGGACTGCTCAAGAAGAAATGGCCCTTTTAGAAGCTGTGATGGACTGTGGCTTTGGAAATTGGCAGGATGTAGCCAATCAAATGTGCACCAAGACCAAGGAGGAGTGTGAGAAGCACTATATGAAGCATTTCATCAATAACCCTCTGTTTGCATCTACCCTGCTGAACCTGAAACAAGCAGAGGAAGCAAAAACTGCTGACACAGCCATTCCATTTCACT |
| si_circRNA_102049_1 | ATTCCATTTCACTACTTCAGAdTdT |
| si_circRNA_102049_2 | TCCATTTCACTACTTCAGATTdTdT |
| hsa-miR-526b-5p mimic | CUCUUGAGGGAAGCACUUUCUGU  GAGAACUCCCUUCGUGAAAGACA |
| hsa-miR-214-3p mimic | ACAGCAGGCACAGACAGGCAGU  UGUCGUCCGUGUCUGUCCGUCA |
| hsa-miR-3619-5p mimic | UCAGCAGGCAGGCUGGUGCAGC  AGUCGUCCGUCCGACCACGUCG |
| hsa_circRNA_102049 | Forward: GCAGAGGAAGCAAAAACTGC  Reverse: CCAATTTCCAAAGCCACAGT |
| hsa-miR-526b-5p | CUCUUGAGGGAAGCACUUUCUGU |
| hsa-miR-214-3p | ACAGCAGGCACAGACAGGCAGU |
| hsa-miR-3619-5p | UCAGCAGGCAGGCUGGUGCAGC |
| GAPDH | Forward: GGAGCGAGATCCCTCCAAAAT  Reverse: GGCTGTTGTCATACTTCTCATGG |
| β-actin | Forward: CACCAGCATCTTTTCCAACC  Reverse: AAGGCCGACTCTCCTACACA |
